# Application of continuous composite RF pulses as components of a fat-suppressed T2-preparation module for 3 Tesla - evaluation of its fat suppression efficiency in clinical cardiac patients

**DOI:** 10.1186/1532-429X-15-S1-P55

**Published:** 2013-01-30

**Authors:** Panki Kim, Elizabeth Jenista, David Wendell, Stephen Darty, Denise Morell, Brenda Hayes, Christoph J Jensen, Whal Lee, Raymond J Kim, Wolfgang G Rehwald

**Affiliations:** 1 Siemens Healthcare, Chapel Hill, NC, USA; 2 DCMRC, Duke University Medical Center, Durham, NC, USA; 3 Seoul National University, Seoul, Republic of Korea

## Background

In myocardial T2-imaging, T2-preparation (T2P) is a common mechanism of creating T2-contrast to reveal pathophysiology. Because T2-contrast is often subtle, bright fat signal can hamper image analysis. Combining T2P with fat suppression (FS) is thus advantageous, but fat saturation is frequently inefficient, and fat inversion by Spectral Attenuated Inversion Recovery (SPAIR) requires high power and constrains sequence timing. We created a T2P module with integrated fat inversion by applying the novel concept of continuous composite RF pulses to create tip-down and flip-back components. We compared this module to two existing modules. Our aim was to develop a shorter T2P compatible FS requiring less power and allowing more flexible timing than SPAIR, but with equivalent suppression efficiency.

## Methods

**Figure 1:**
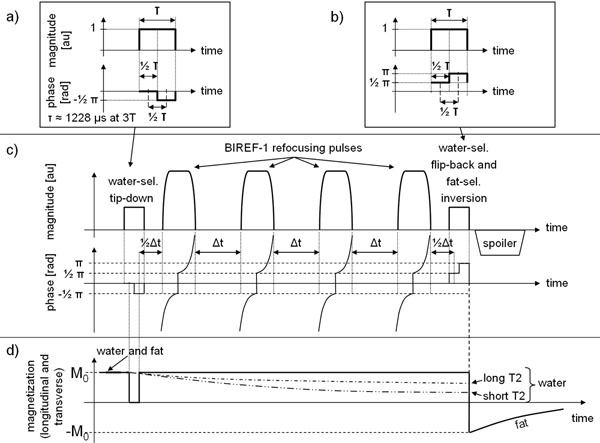
a) Continuous composite water-selective tip-down: The first half of the pulse rotates water and fat magnetization into the transverse plane; the second half flips only fat back. A 90° fat/water shift exists between the center of the first and the center of the second (τ/2 = 614 µs). The pulse causes two consecutive rotations about the x and the -y axis. b) Continuous composite water-selective flip-back and fat-selective inversion. The phase modulation corresponds to a rotation about y for the first half and about -x for the second. c) The novel T2PFS module using above pulses and four B1 insensitive refocusing pulses (BIREF-1). Time ½Δt is measured from the end of the refocusing pulse to the first quarter of the continuous composite pulse. d) Relaxation curves of water and fat showing T2-weighting of water and inversion of fat magnetization. Transverse and longitudinal relaxation processes are combined in one graph.

**Figure 2:**
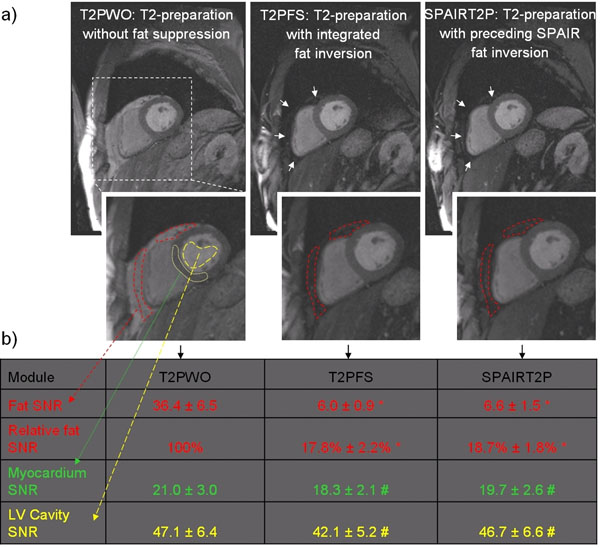
a) Typical T2-prepared patient images obtained with the three modules. Note the excellent suppression of pericardial fat by T2PFS and SPAIRT2P, see white arrows. Signal-to-noise ratio (SNR) was measured in fat (red dashed ROIs), myocardium (green dotted ROI), and LV cavity (yellow dashed ROI). Typical sequence parameters were trigger pulse 2, fov 360 x 270 mm, matrix 256 x 125, segments 21, flip angle 15°, receiver bandwidth 399 Hz/pixel, TE 1.66 ms, TR 4.4 ms, slice thickness 6 mm. b) Table lists SNR mean and SNR standard error of the mean measured in 11 patients for the three modules in the indicated ROIs. *: statistically different from T2PWO (p < 0.001). #: statistically identical to T2PWO (p > 0.05).

Our tip-down and flip-back components are continuous composite RF pulses, each derived as a two-part composite pulse, but played as a single continuous pulse. Designing the pulse duration and a separate phase modulation for each part allows an independent rotation of water and fat. The tip-down (Figure 1a) is water-selective. The flip-back (Figure 1b) rotates water to the z-axis and simultaneously inverts fat. Sets of 3 images were acquired in 11 cardiac patients on a 3T Siemens MAGNETOM Verio MR scanner. Parameters were identical except for the T2P module: 1) T2P without FS and standard rectangular tip-down/flip-back pulses (T2PWO), 2) T2P with integrated fat inversion (our new module, T2PFS, Figure 1c), and 3) T2P preceded by a SPAIR pulse for fat inversion (SPAIRT2P). SNR was measured in 2 peri- or epicardial fat regions of interest (ROIs), in LV cavity and in myocardium (Figure 2a). To quantify FS efficiency, fat SNR of T2PFS and SPAIRT2P was normalized to fat SNR of T2PWO in the same ROI and expressed as relative SNR (%). Myocardial and cavity SNR were measured to evaluate if T2PFS or SPAIRT2P affected tissue and blood SNR. An ANOVA with Bonferroni correction was applied to test for statistical differences between groups T2PWO, T2PFS, and SPAIRT2P in fat, myocardium, and cavity. Module energy was also calculated.

## Results

The designed T2PFS inverts fat at the end of the module while simultaneously providing T2-weighting of water (Figure 1d). Figure 2a shows typical T2-prepared patient images using T2PWO, T2PFS, and SPAIRT2P. Visual inspection revealed excellent suppression of pericardial fat by T2PFS and SPAIRT2P (arrows). Statistical analysis of relative SNR confirmed significantly suppressed fat by T2PFS and SPAIRT2P, while myocardium and cavity SNR were not affected compared to T2PWO (Figure 2b). Typical energy of T2PWO, T2PFS, and SPAIRT2P was 148.0 Ws, 152.0 Ws and 153.9 Ws, respectively. Energy per fat inversion by T2PFS was 30.5% lower than by SPAIRT2P.

## Conclusions

We present a novel T2P with integrated fat inversion that is shorter and requires less power than SPAIR, but with the same excellent FS efficiency as SPAIR.

## Funding

None

